# An Efficient, Versatile, and Safe Access to Supported Metallic Nanoparticles on Porous Silicon with Ionic Liquids

**DOI:** 10.3390/ijms17060876

**Published:** 2016-06-03

**Authors:** Walid Darwich, Paul-Henri Haumesser, Catherine C. Santini, Frédéric Gaillard

**Affiliations:** 1 CNRS-Université de Lyon-ESCPE Lyon, UMR 5265 C2P2, 43 Bd du 11 Novembre 1918, Villeurbanne 69616, France; darwich.walid@hotmail.com (W.D.); catherine.santini@univ-lyon1.fr (C.C.S.); 2 CEA, LETI, MINATEC Campus, Grenoble 38054, France; 3 Université Grenoble Alpes, Grenoble 38000, France; frederic-x.gaillard@cea.fr

**Keywords:** porous silicon, metallization, surface reactivity, metallic nanoparticles

## Abstract

The metallization of porous silicon (PSi) is generally realized through physical vapor deposition (PVD) or electrochemical processes using aqueous solutions. The former uses a strong vacuum and does not allow for a conformal deposition into the pores. In the latter, the water used as solvent causes oxidation of the silicon during the reduction of the salt precursors. Moreover, as PSi is hydrophobic, the metal penetration into the pores is restricted to the near-surface region. Using a solution of organometallic (OM) precursors in ionic liquid (IL), we have developed an easy and efficient way to fully metallize the pores throughout the several-µm-thick porous Si. This process affords supported metallic nanoparticles characterized by a narrow size distribution. This process is demonstrated for different metals (Pt, Pd, Cu, and Ru) and can probably be extended to other metals. Moreover, as no reducing agent is necessary (the decomposition in an argon atmosphere at 50 °C is fostered by surface silicon hydride groups borne by PSi), the safety and the cost of the process are improved.

## 1. Introduction

Porous silicon (PSi) has been attracting a great amount of attention as a breakthrough material with exceptional characteristics for microelectronics, integrated optoelectronics, microelectromechanical systems (MEMS), layer transfer technology, solar and fuel cells, biomedicine, *etc*. [1,2]. Depending on the conditions for its elaboration, PSi can exhibit a wide range of morphological properties, from extremely small pores below 5 nm (microporous) to micrometric pores (macroporous). Moreover, its high surface area (microporous silicon ~1000 m^2^/cm^3^, mesoporous silicon ~100 m^2^/cm^3^, macroporous silicon ~1 m^2^/cm^3^) makes it suitable to host one or more guest materials which usually results in a drastic change of its physical properties [2]. For instance, the addition of a well-chosen metal can provide specific composite structures with new electrical, optical, magnetic, plasmonic, or other properties [3]. In particular, the introduction of Cu improves the optoelectronic functions of PSi, e.g., its photoluminescence and electroluminescence [4].

The metal can be incorporated by electrochemical or electroless methods, but also by physical evaporation. However, upon evaporation or sputtering, metal atoms remain localized at the entrance of pores [3]. By contrast, immersion methods enhance the penetration of metal atoms deeper into the porous layer [5,6].

Importantly, PSi can reduce many metal ions down to their elemental state. This is due to the presence of SiH_x_ bonds formed during the electrolytic synthesis of the material. This can in principle enable the deposition of various noble and coinage metals in the porous layer by a chemical surface reaction [7,8,9,10,11].

In aqueous solutions, the source of electrons needed to reduce z-charged metal ions is PSi itself [12].

(1) $$4M^{z+}+\text{zSi}+2{\text{zH}}_{2}O\to 4M+{\text{zSiO}}_{2}+4{\text{zH}}^{+}$$

However, the hydrophobic inner pore walls are impermeable to water contributing to concentrate metal deposits on the surface in patches rather than as a conformal layer down into the pores [7,8,12,13].

Nevertheless, this approach remains attractive and elegant, as it could provide an easy, low-cost, and versatile process to metallize PSi. Hence, the purpose of this study is to identify a liquid medium:

1. Capable of penetrating the diverse porous structures [14];
2. in which different metal precursors are soluble [15,16];
3. that does not prevent the reaction of these precursors with surface hydrides [17];
4. and in which additional reagents can be added to extend metal precursor decomposition [15,18].

We believe that ionic liquids (ILs) fulfill all these requirements. Indeed, ILs are suitable media to solubilize a wide range of metal precursors. From these solutions, zero-valent metallic nanoparticles (NPs) are easily precipitated using appropriate reducing agents such as H_2_ [19,20,21,22]. Moreover, ILs penetrate quite rapidly in porous materials [14]. Therefore, we investigate in this work the ability of imidazolium-based ILs containing organometallic (OM) precursors to metallize a variety of micro- to macro-porous silicon substrates.

## 2. Results and Discussion

### 2.1. Surface Chemistry of PSi Substrates

Two different porous Si materials were electrochemically etched in p-doped Si wafers (resistivity of 1500 Ohm.cm): macroporous Si (MPSi) and microporous Si (µPSi). Their morphological characteristics are listed in Table 1. Note that, within the macropores of MPSi, portions of the internal surface are microporous. Samples of bulk Si (BSi) were considered as a reference throughout this study.

**Figure 1:**
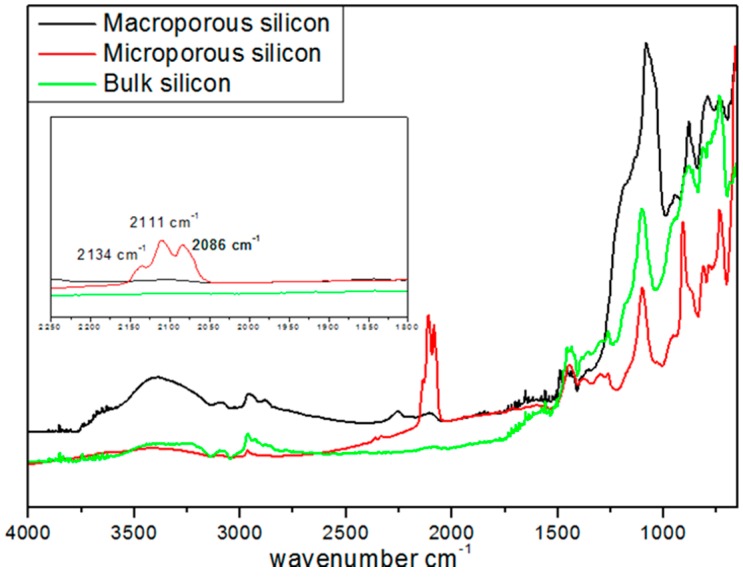
DRIFT spectra of macroporous Si (MPSi), microporous Si (µPSi), and bulk Si (BSi) samples in an argon atmosphere at 50 °C.

Before use, these substrates were dried in a strong vacuum (10^−5^ bar, overnight) and stored in a glove box in an argon atmosphere. Diffuse reflectance infra-red Fourier transform analysis (DRIFT) was used to characterize the surface chemical groups of these various substrates (Figure 1).

For all the samples, the peaks at 1100 and at 3500 cm^−1^ were assigned to ν(≡Si-O-Si≡) and ν(≡Si-OH) vibrations, respectively. The peaks at 2134, 2111, 2086, and 2254 cm^−1^ were attributed to ν(SiSiH_3_), ν(Si_2_SiH_2_), ν(Si_3_SiH), and ν(O_3_SiH) vibrations, respectively. The latter peaks were not detected in BSi and exhibited a much lower intensity in MPSi as compared to µPSi. This difference of surface silicon hydride concentration between the two porous materials was confirmed by temperature-programmed desorption experiments performed between 100 and 900 °C. A total of 0.628 mmol·g^−1^ of H_2_ was released by µPSi *vs.* 0.055 mmol·g^−1^ of H_2_ for MPSi [23].

### 2.2. Metallization of PSi

#### 2.2.1. Metallization from a Suspension of Metallic NPs in IL

Suspensions of well-dispersed crystalline metallic zero-valent Cu-NPs of 5 nm with a narrow size distribution were routinely synthesized from the reduction of mesitylcopper (CuMes) in 1-butyl-3-methylimidazolium bis(trifluoromethylsulphonyl)imide [C_1_C_4_Im][NTf_2_] [24]. These stable Cu-NP suspensions could be promising starting materials to achieve conformal copper deposition into PSi layers.

**Figure 2:**
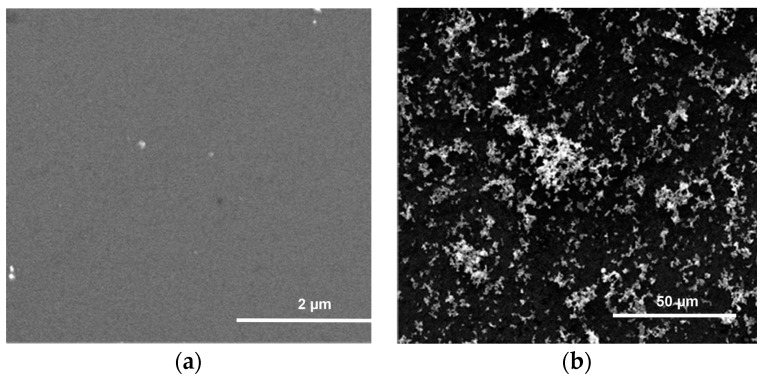
Surface of (**a**) BSi and (**b**) µPSi samples after treatment through the *ex-situ* procedure.

**Figure 3:**
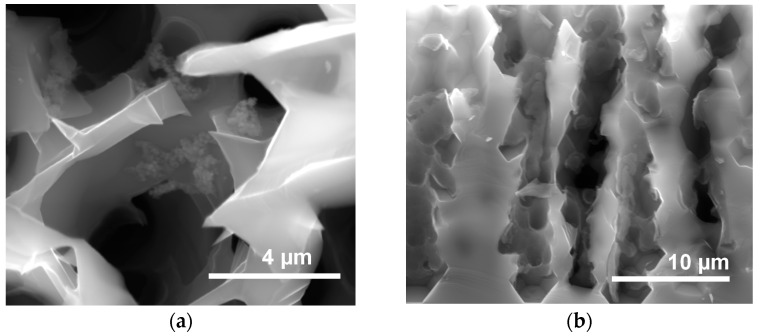
MPSi sample tentatively metallized through the *ex-situ* procedure. (**a**) Cu aggregates are visible on the surface; (**b**) but not in the pores.

BSi and PSi substrates were impregnated with a suspension of 5 nm Cu-NPs in [C_1_C_4_Im][NTf_2_] in an argon atmosphere for 2 h at 50 °C, an *ex-situ* procedure. After treatment, the samples were rinsed with CH_2_Cl_2_. No Cu deposition was observed on the BSi surface (Figure 2a). This indicates that surface silanol ≡Si-OH are inefficient for the grafting of Cu-NPs. The metal was washed away via rinsing with CH_2_Cl_2_ and subsequently dried. By contrast, MPSi and µPSi samples exhibited large Cu aggregates with a broad size distribution on their surface (Figure 2b and Figure 3a). Such a morphology could result from the agglomeration of the weakly adherent Cu-NPs during the rinsing and drying processes [25]. This could mean that surface hydrides are needed to graft Cu-NPs. This could also be related to the rougher topography of these substrates. Nevertheless, no Cu was detected in their pores (Figure 3b). This can be expected in µPSi (the pores have the same size as the NPs), but is more surprising for MPSi. Finally, this procedure proves not to be efficient to metallize the internal surface of the pores [23].

#### 2.2.2. Metallization from a Solution of CuMes in IL

**Figure 4:**
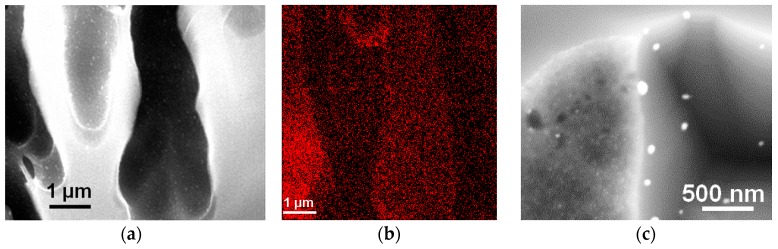
MPSi sample metallized by decomposing CuMes in [C_1_C_4_Im][NTf_2_]. (**a**) Cu islands are clearly visible in the pores; as confirmed by (**b**) EDX mapping of Cu; (**c**) Cu is detected within the hollow macropores. The highest Cu concentration was detected at the pore walls near the cross-section plane.

In the first experiment, a MPSi substrate was placed in an autoclave and impregnated by a solution of CuMes in [C_1_C_4_Im][NTf_2_] under argon atmosphere for 2 h at 50 °C. The autoclave was then placed under 0.9 MPa H_2_, for 4 h at 100 °C. Interestingly enough, this procedure afforded Cu islands, both at the surface and in the pores, after a rinsing process (Figure 4a). Electron dispersive X-ray analysis (EDX) mapping (Figure 4b) confirms that Cu is coating the inner walls of the macropores. The highest Cu concentration was detected at the pore walls near the cross-section plane (red). A closer examination of the morphology of the Cu deposit shows that it was formed of large (>150 nm) Cu islands on the flat walls of the pores and smaller ones (about 12 nm) on the microporous walls of macropores (Figure 4c). This could be related to the difference in roughness (rougher surfaces induce more nucleation) or to the increased concentration of surface silicon hydride specifically in the micropores.

**Figure 5:**
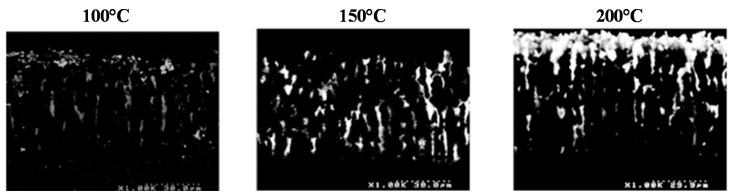
MPSi samples metallized by decomposition of a solution of CuMes in IL at 100, 150 and 200 °C observed using the back-scattered electron detector (BSED) of the SEM.

In an attempt to increase metal content in the MPSi layer, similar experiments were performed at higher temperatures (150 and 200 °C). The back-scattered electron (BSED) scanning electron microscopy (SEM) images in Figure 5 show that, upon increasing temperature to 150 °C, more Cu was indeed detected throughout the porous material. At 200 °C, however, Cu was preferentially deposited near the surface, blocking access to the pores below. The reaction was probably too fast and did not allow for the diffusion of CuMes down to the bottom of the porous layer.

Finally, MPSi is a complex material which incorporates planar surface portions as well as micropores. These different surfaces bear different amounts of surface silanol and hybride groups, which are probably involved in either the NP formation or their grafting. To separate these factors, two reference silicon samples can be considered: BSi, which exhibits no porosity and only silanol surface groups (Figure 1), and μPSi, containing only micropores in which surface silicon hydride groups dominate. Moreover, the latter material is best suited to the spontaneous Cu deposition upon immersion in solutions containing cupric ions because of its reductive character [5,26,27,28].

**Figure 6:**
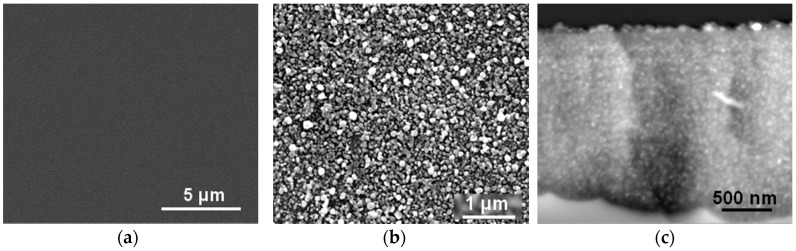
(**a**) Top-view SEM image of BSi surface; (**b**) top-view and (**c**) cross section SEM images (using backscattering diffusion detector) of µPSi sample after impregnation by a solution of CuMes in [C_1_C_4_Im][NTf_2_] under argon atmosphere for 2 h at 50 °C.

In order to verify if this reactivity of µPSi still holds in IL-based solutions, the metallization of μPSi and BSi was attempted by sole impregnation with a solution of CuMes in [C_1_C_4_Im][NTf_2_] under argon atmosphere for 2 h at 50 °C. Interestingly enough, after rinsing with CH_2_Cl_2_, an almost closed Cu film remained on the µPSi surface (Figure 6b). Even more interestingly, Cu islands with a diameter of about 10 nm also coated the inner surface of the pores (Figure 6c). Hence, CuMes is indeed readily decomposed by µPSi. In contrast, no Cu deposit remained at the surface of BSi (Figure 6a). This rules out the oxidation of Si as the second half-reaction responsible for metal deposition in water. Furthermore, these results indicate that the surface hydride groups decompose the Cu precursor and anchor the Cu-NPs. This would explain why different sizes and surface coverage of Cu islands are observed in the flat *vs.* microporous internal surfaces of MPSi. In conclusion, this easy procedure allows for the metallization by Cu of a variety of porous Si samples without H_2_ [23].

### 2.3. Generalization to Other Metals

As highlighted in the introduction, this procedure should be suited to deposit other metals as well. To explore this possibility, this process was applied to solutions of other OM precursors in IL.

#### 2.3.1. Palladium

**Figure 7:**
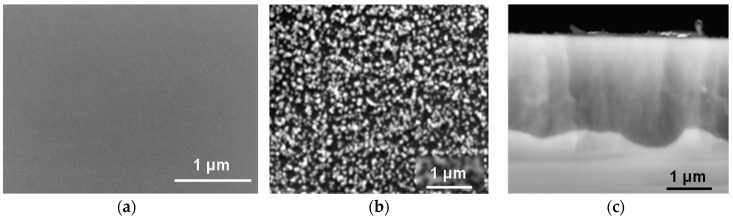
SEM images after reaction with Pd(dba)_2_ in [C_1_C_4_Im][NTf_2_] at 50 °C under argon atmosphere for 2 h. (**a**) BSi surface; µPSi (**b**) surface and (**c**) micropores (cross section).

BSi and µPSi samples were treated with a solution of Pd(dba)_2_ in [C_1_C_4_Im][NTf_2_] for 2 h at 50 °C under argon atmosphere. As expected, no Pd deposit was observed on the BSi surface (Figure 7a). By contrast, a population of large Pd islands was present at the surface of µPSi (Figure 7b) and a dense population of Pd-NPs (12 nm in diameter) in the micropores (Figure 7c). These results are thus similar to those obtained with CuMes, indicating that the same mechanisms are at play.

#### 2.3.2. Platinum

**Figure 8:**
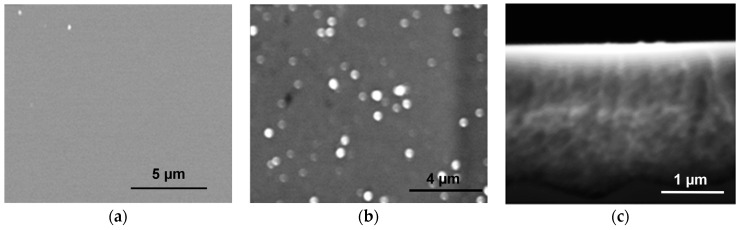
SEM images after reaction with Pt(dba)_2_ in [C_1_C_4_Im][NTf_2_] at 50 °C under argon atmosphere for 2 h. (**a**) BSi surface; µPSi (**b**) surface and (**c**) micropores (cross section).

With Pt(dba)_2_ as an OM precursor, no Pt deposit formed on the (BSi) surface (Figure 8a), whereas populations of large Pt islands and small particles were observed on the µPSi surface and in the micropores, respectively (Figure 8b,c). The EDX analysis confirms the presence of Pt with some traces of ILs. Note that the overall amount of deposited Pt is weaker than for Pd and Cu. This could be ascribed to (i) the larger size of Pt particles on the surface (≈400 nm *vs.* ≈40 nm for Cu and Pd) and (ii) a partial leaching of the Pt-NPs during the rinsing process (the anhydrous CH_2_Cl_2_ went from colorless to red amid rinsing).

**Figure 9:**
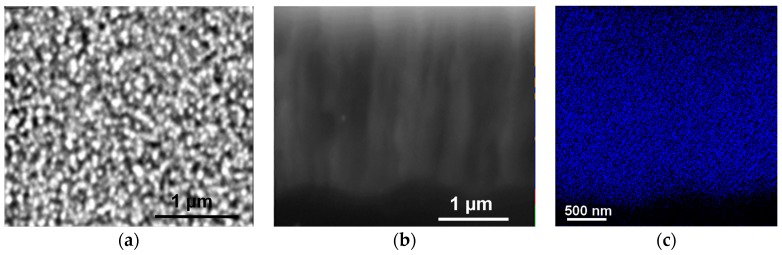
SEM images of (**a**) the surface (**b**) the cross-section of µPSi after reaction with Pt(Me)_2_(COD) in [C_1_C_4_Im][NTf_2_] at 50 °C under argon atmosphere for 2 h; (**c**) EDX mapping of Pt in the cross section.

To increase Pt content in µPSi, the more reactive (COD)Pt(Me)_2_ was tested. With this precursor, the density and size of Pt-NPs were similar to those of Cu and Pd (Figure 9). However, the metal concentration decreased with depth. As explained with high-temperature experiments with CuMes (see Figure 5), this can be ascribed to an overly fast decomposition of (COD)Pt(Me)_2_, as compared to the rate of penetration of the precursor into the pores.

#### 2.3.4. Ruthenium

**Figure 10:**
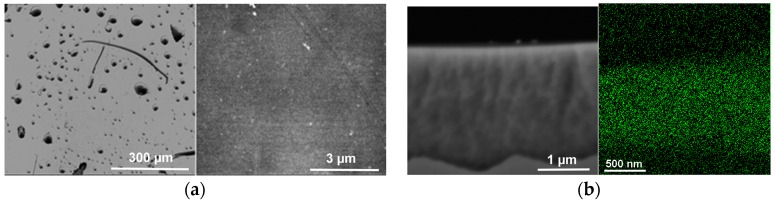
SEM images of the surface of µPSi after reaction with (COD)Ru(2-methylallyl)_2_ in [C_1_C_4_Im][NTf_2_] at 50 °C under argon atmosphere for 2 h and after washing. (**a**) Left twice or right five times with CH_2_Cl_2_; (**b**) cross section of µPSi and corresponding EDX mapping of Ru.

With (COD)Ru(2-methylallyl)_2_, it was found that rinsing with CH_2_Cl_2_ resulted in a dark solution. Several successive rinses were needed to obtain a clear solution and properly remove residual IL from the surface. Therefore, in this case, significant leaching of Ru (RuNPs or starting material) occurred. As a result, only sparse particles remained on the μPSi surface (Figure 10a). Nevertheless, RuNPs were still present in the pores, as confirmed by SEM and EDX mapping (Figure 10b).

## 3. Materials and Methods

All operations were performed in the strict absence of oxygen and water under purified argon atmosphere using glove-box (MBraun) or vacuum-line techniques. Bis(dibenzylideneacetone)platinum, Pt(dba)_2_, Bis(dibenzylideneacetone)palladium(0) (Pd(dba)_2_) (Strem), (1,5-cyclooctadiene)dimethylplatine(II) (Pt(Me)_2_(COD)) (Nanomeps), bis(2-methylallyl)(1,5-cyclooctadiene)ruthenium(II) (Ru(2-methylallyl)_2_(COD)) (Sigma Aldrich, St. Louis, MO, USA) and mesitylcopper (CuMes) (Nanomeps) were kept in a refrigerator in the glove box and used as received.

1-Butyl-3-methylimidazoliumbis(trifluoromethylsulphonyl)imide, [C_1_C_4_Im][NTf_2_], was prepared as already reported [29]. Its purity was checked via NMR spectra recorded on a Bruker Advance spectrometer at 300 MHz for ^1^H and at 75.43 MHz for ^13^C. After purification, the halide content was found to be below 100 ppm (HR-SM), and water found to be ~12 ppm (limit of Karl Fischer titration).

The solutions were freshly prepared by dissolving CuMes in [C_1_C_4_Im][NTf_2_] to the desired concentration (5 × 10^−2^ mole·L^−1^) in a Schlenk tube under stirring at room temperature.

Suspensions of Cu-NPs were prepared as already reported [24]. The NPs were observed by TEM using a Philips CM120 at 120 kV. For this purpose, in the glove box, the suspensions were deposited on a TEM grid and transferred into the microscope without further preparation. For each suspension, the size of at least 200 NPs was measured. Their size distribution was then fitted by a lognormal law.

Different types of Si surfaces were used—two porous and one non-porous (bulk) surface as a reference. The porous layers were electrochemically generated in a hydrofluoric solution on 200-mm Si wafers using an automated piece of equipment composed of a cleaning and an anodizing chamber.

## 4. Conclusions

An original process for the metallization of porous Si (PSi) materials was investigated here. Ionic liquids were shown to be efficient in impregnating PSi layers and to convey organometallic (OM) precursors at the bottom of the pores. Interestingly enough, microporous Si (µPSi), which is known to reduce metal ions in aqueous solutions, is able to decompose OM precursors in ILs as well. However, this reaction most probably involves the surface hydride groups, in contrast with aqueous media (in which Si is oxidized). In addition, these groups contribute to the grafting of the metal. The reduction of CuMes by these surface hydride groups is still under investigation. However, it has been successfully generalized to other metals such as Pt, Pd, and Ru. Finally, this approach does not require additional reducing agents; hence, the safety and the cost of the process are improved.

## Figures and Tables

**Table 1:** Samples used in this study.

Material	Porous Layer	
	Pore Diameter	Thickness
Macroporous Si (MPSi)	5 µm (+5 nm)	20 µm
Microporous Si (µPSi)	5 nm	2 µm

## Abbreviations

IL	ionic liquids
NP	nanoparticle
[C_1_C_4_Im][NTf_2_]	1-butyl-3-methylimidazolium bis(trifluoromethylsulphonyl)imide
SEM	scanning electron microscope
EDX	energy-dispersive X-ray spectroscopy
